# An Overview of the Isoprenoid Emissions From Tropical Plant Species

**DOI:** 10.3389/fpls.2022.833030

**Published:** 2022-05-20

**Authors:** Zhaobin Mu, Joan Llusià, Jianqiang Zeng, Yanli Zhang, Dolores Asensio, Kaijun Yang, Zhigang Yi, Xinming Wang, Josep Peñuelas

**Affiliations:** ^1^State Key Laboratory of Organic Geochemistry and Guangdong Key Laboratory of Environmental Protection and Resources Utilization, Guangzhou Institute of Geochemistry, Chinese Academy of Sciences, Guangzhou, China; ^2^CAS Center for Excellence in Deep Earth Science, Guangzhou, China; ^3^CSIC, Global Ecology Unit CREAF-CSIC-UAB, Barcelona, Spain; ^4^CREAF, Barcelona, Spain; ^5^College of Resources and Environment, University of Chinese Academy of Sciences, Beijing, China; ^6^Center for Excellence in Regional Atmospheric Environment, Institute of Urban Environment, Chinese Academy of Sciences, Xiamen, China; ^7^Fujian Provincial Key Laboratory of Soil Environmental Health and Regulation, College of Resources and Environment, Fujian Agriculture and Forestry University, Fuzhou, China

**Keywords:** BVOCs, isoprenoids, tropical species, emission inventory, emission variations, emission models

## Abstract

Terrestrial vegetation is the largest contributor of isoprenoids (a group of biogenic volatile organic compounds (BVOCs)) to the atmosphere. BVOC emission data comes mostly from temperate regions, and less is known about BVOC emissions from tropical vegetation, even though it is estimated to be responsible for >70% of BVOC emissions. This review summarizes the available data and our current understanding of isoprenoid emissions from tropical plant species and the spatial and temporal variation in emissions, which are strongly species-specific and regionally variable. Emission models lacking foliar level data for tropical species need to revise their parameters to account for seasonal and diurnal variation due to differences in dependencies on temperature and light of emissions from plants in other ecosystems. More experimental information and determining how emission capacity varies during foliar development are warranted to account for seasonal variations more explicitly.

## Introduction

A diverse array of volatile and semi-volatile chemicals is emitted by terrestrial vegetation (Goldstein and Galbally, 2007; Guenther et al., 2012), which is by far the largest contributor to the global annual flux of reactive volatile organic compounds to the atmosphere (Lamarque et al., 2010; Guenther et al., 2012). Emissions of BVOCs from vegetation account for 90% of total global non-methane VOCs (NMVOCs; Guenther et al., 1995; Singh et al., 2011). Isoprenoids have the highest rates of emission and the greatest atmospheric impact (Guenther et al., 1995; Kuhn et al., 2004b) among BVOCs. Their emissions are highly dependent on land cover, including the composition and dominance of plant species and the environmental conditions that influence species physiology (Guenther et al., 1995; Taylor et al., 2018, 2019). BVOC emissions from terrestrial vegetation have been studied for several decades because they play important roles in atmospheric chemistry and plant physiology (Went, 1960; Rasmussen, 1972). On the one hand, they strongly affect atmospheric composition by contributing to the photochemical production of ozone and the formation of secondary particles (Fehsenfeld et al., 1992; Wang et al., 2007; Singh et al., 2014), thereby influencing regional air quality and climate (Peñuelas and Llusià, 2003; Derwent et al., 2007; Peñuelas and Staudt, 2010; Guenther et al., 2012). On the other hand, there are BVOCs that can mediate plant–plant, plant–insect, and plant–microbe interactions (Peñuelas and Llusià, 2001; Kishimoto et al., 2006; Gershenzon and Dudareva, 2007; Peñuelas and Staudt, 2010; Jardine et al., 2015; Maag et al., 2015) and protect photosynthetic membranes against abiotic stresses (Peñuelas and Llusià, 2002; Vickers et al., 2009; Jardine et al., 2017; Yàñez-Serrano et al., 2018).

Although the tropics are estimated to emit >70% of the global total BVOC emissions (Karl et al., 2007; Jones et al., 2011), the number of investigations there has been heavily limited by complexity in eco-regional variety and species diversity, as well as logistical and methodological difficulties (Geron et al., 2002; Kuhn et al., 2002, 2004a,b; Harley et al., 2004; Bracho-Nunez et al., 2013). These disadvantages also hinder our understanding of the chemistry of the lower troposphere over the tropics (Harley et al., 2004; Kuhn et al., 2004a; Paton-Walsh et al., 2022). Furthermore, it should be added that the magnitude and chemical speciation of BVOC emissions of vegetation are highly species-specific (Wang et al., 2007) and vary with environmental conditions and the developmental stages of plants (Lerdau and Throop, 2000; Kuhn et al., 2004a; Wilske et al., 2007). Studies of species-level BVOC emissions that provide the emission factor measured at the leaf level under standard conditions (leaf temperature of 30°C and photosynthetically active radiation (PAR) of 1,000 μmol m^−2^ s^−1^; Guenther et al., 1993; Monson et al., 2012) are essential for developing regional models that can be used to predict emissions across large spatial and temporal scales (Lerdau and Throop, 2000).

The primary aim of this paper is to compile a BVOC emission factor inventory of tropical species and to explore their basic emission characteristics. We collected and analyzed available data for isoprenoid emissions from tropical plants for all tropical and some subtropical regions (30°N to 30°S; Appendix S1). We discuss our current understanding of the effects on BVOC emissions of the diurnal and seasonal variations due to environmental and physiological factors. Finally, we review studies of emission models and techniques for measurement used for tropical species. The scope of this review is to summarize the dynamics determining the variation in BVOC emission capacity across space and time in tropical landscapes. Compared to the recent comprehensive review of BVOCs from the Amazonia (Yáñez-serrano et al., 2020), our review is differentiated by having an emission factor inventory across larger tropical landscapes and a more specific focus toward updating the tropical emission algorithms.

## Profile of Isoprenoid Emissions From Tropical Species

**Figure 1:**
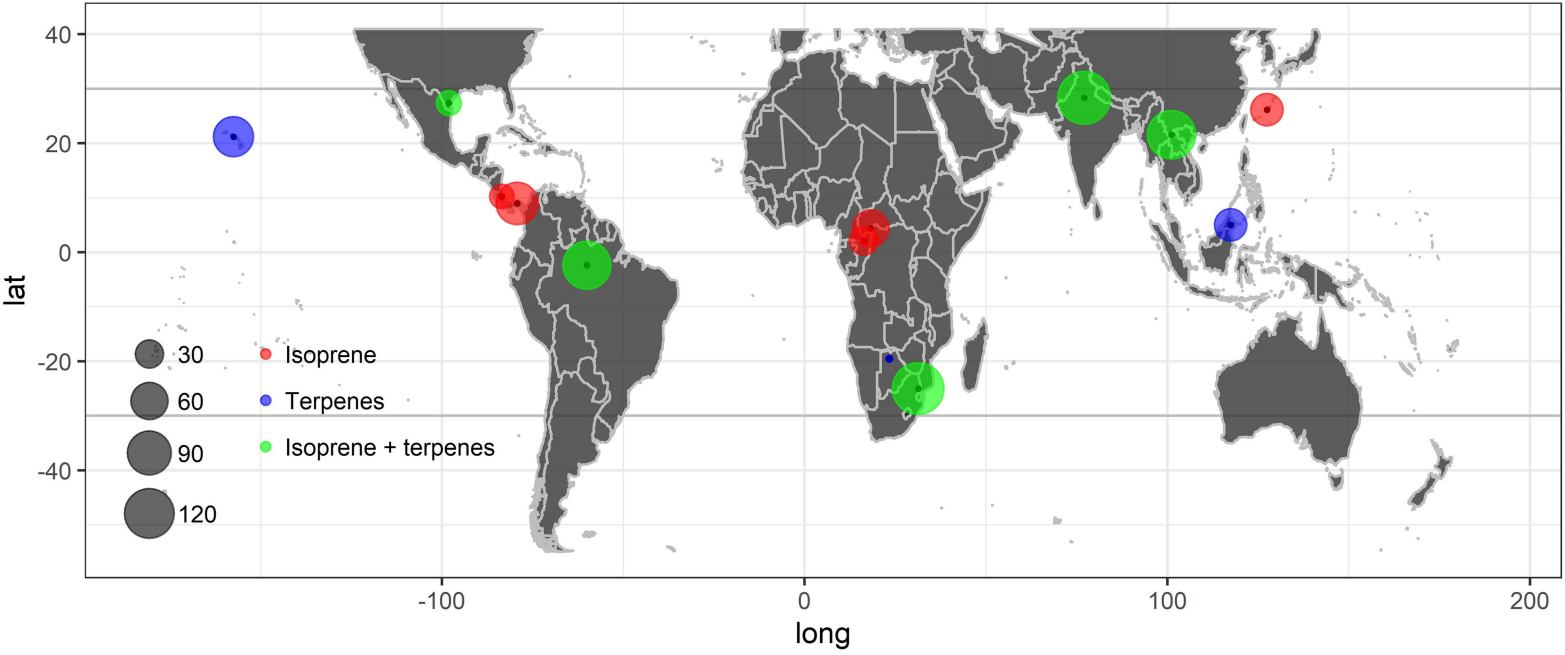
Distribution of the tropical plant species with isoprenoid emissions reviewed in this study (Appendix S1). Red represents areas where isoprene is the only isoprenoid reported, blue represents areas where terpenes are the only isoprenoids reported, green represents areas where isoprene, terpenes, or both isoprene and terpenes are the isoprenoids reported. The size of the circle is proportional to the number of tropical species studied.

BVOC emissions released by tropical plant species have been studied most in Brazil (South America), India and China (Asia), and the Republic of South Africa (Africa; Figure 1) among subtropical and tropical regions, where more than 100 species were studied taking both emitted isoprene and terpenes into consideration (Figure 1; Appendix S1). Isoprene was the main compound, which was emitted by 76.1% (83 of 109) of the species studied in Brazil, 73.5% (100 of 136) in India, 58.3% (63 of 108) in China, and 53.8% (70 of 130) in the Republic of South Africa. Isoprene may thus be emitted by more than half of all tropical terrestrial species, while the neotropics represented by Amazonian regions may have larger proportions of emitters compared to other tropics. There is a large proportion of emitters among tropical species, as emitting species outperform non-emitting species by assimilating more carbon at high temperatures (Taylor et al., 2019). Meanwhile, this coincided with the previous inference that isoprene is likely to be a key compound of vegetation in response to environmental stresses and climate change, especially in tropical forests emitting more isoprene than other ecosystems (Guenther et al., 2006; Taylor et al., 2018). However, the distributions of isoprene-emitting species on the landscape vary quantitatively with climatic factors. For example, Taylor et al. (2018) provided explicit estimates of the proportion of emitting trees, which increases with mean annual temperature and decreases with length of dry season. Notably, the percentages of emitters are much higher than those in previous reports, indicating that more than one-third of species emit isoprene in tropical forests (Harley et al., 2004; Taylor et al., 2018). Furthermore, Jardine et al. (2020) found 61.9% of all 113 investigated dominant species in the Amazon basin emitted isoprene, which is also lower than the 76.1% reported in this study. This may be due to our compilation focused on quantitative measurements of emissions rather than “survey-type” emission inventories; qualitative or semi-quantitative emission potential studies were excluded from our compilation. In addition, this study included larger quantities of plant species from tropical and subtropical landscapes, not only trees growing in forests. However, these inferences still warrant validation through further similar screening studies throughout the world (Llusià et al., 2014).

**Figure 2:**
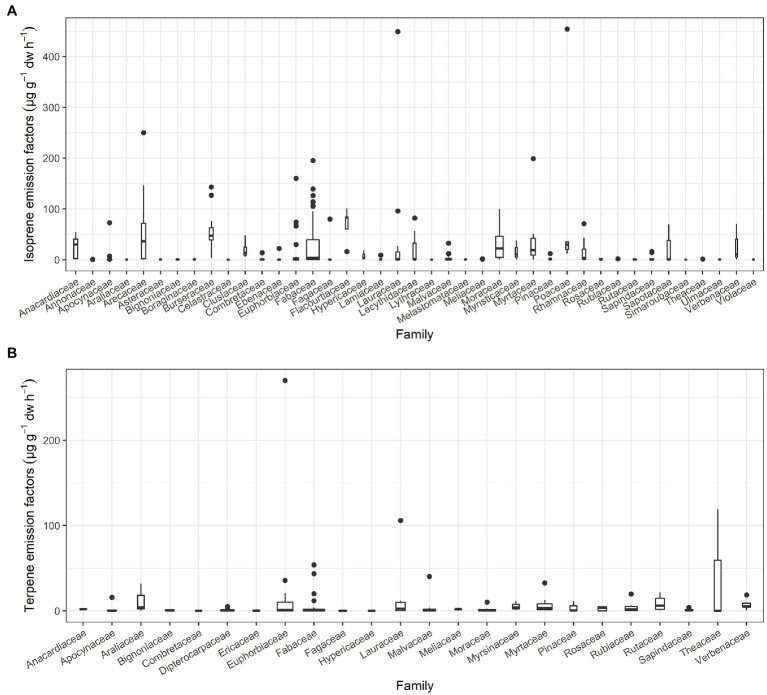
Factors of isoprene **(A)** and terpene **(B)** emissions with more than three species for tropical plant families (Appendix S1). The box is drawn from the first quartile (Q1) with a whisker to the minimum to the third quartile (Q3) with a whisker to the maximum. The median is represented by the line in the box, and outliers are identified by dots. The width of box is proportional to the number of tropical species studied.

We used the foliar level data in μg g^−1^ dw h^−1^ to determine family-level capacity of isoprene emissions (Appendix S1). There were three typical patterns for family-level isoprene emissions in tropical plants. The most important families for isoprene emissions include Anacardiaceae, Arecaceae, Burseraceae, Clusiaceae, Euphorbiaceae, Fabaceae, Flacourtiaceae, Lauraceae, Lecythidaceae, Moraceae, Myristicaceae, Myrtaceae, Poaceae, Rhamnaceae, Sapotaceae, and Verbenaceae. Species in the families Annonaceae, Araliaceae, Asteraceae, Bignoniaceae, Boraginaceae, Celastraceae, Lamiaceae, Lythraceae, Melastomataceae, Meliaceae, Rosaceae, Rubiaceae, Rutaceae, Simaroubaceae, Theaceae, Ulmaceae, and Violaceae were not emitters or very low emitters (Figure 2A; Appendix S1). Families, such as Apocynaceae, Combretaceae, Ebenaceae, Fagaceae, Hypericaceae, Malvaceae, Pinaceae, and Sapindaceae, were also low emitters but contained one or two species that were high emitters.

Studies of terpenes were much less common than those of isoprene and dominated by monoterpenes or monoterpenes combined with sesquiterpenes. Meanwhile, tropical plants emit much less terpenes than isoprene in terms of the amount and capacity of emitters (Figure 2). The most important tropical plant families for terpene emission included Araliaceae, Euphorbiaceae, Fabaceae, Lauraceae, Myrtaceae, Rubiaceae, Rutaceae, Theaceae, and Verbenaceae (Figure 2B; Appendix S1).

These studies have been concentrated mostly in India, China, Malaysia, and the United States (Figure 1; Appendix S1). The Asia-Pacific islands had a similarly high percentage, with 97.2% (70 of 72) of the species studied in Hawaii and 95.3% (41 of 43) in Borneo emitting monoterpenes, but the continents had much lower percentages, with 75.7% (81 of 107) and 47.4% (46 of 97) of the species studied in India and China, respectively. In addition, Llusià et al. (2010) recorded sesquiterpenes from 68.1% (49 of 72) of the species studied in Hawaii to 81.4% (35 of 43) and in Borneo (Llusià et al., 2014). At the same time, these authors found that there are variations in terpene emission capacity between exotic and native species. The rates of total terpene emission were 1.5-fold higher in exotic than native species (*p* < 0.05) and only coincided with a significant difference in α-pinene (*p* < 0.05), which was more than twice as high in exotic species in Hawaii (Llusià et al., 2010), while the rates of isoprene emission from 42 tropical tree species did not differ significantly between exotic and native species in Okinawa (Tambunan et al., 2006). These findings suggest that invasive species may benefit from a higher terpene emission rate, eventually by being better protected against multiple stresses and that the higher terpene emission may partly account for their acclimation in the new environment, which may also imply that invasive species may respond more competitively to new combined stresses driven by global change in tropical regions (Llusià et al., 2010; Loreto and Fineschi, 2015). Meanwhile, islands may be much more subject to species invasion than inland continents (Bellard et al., 2017; Dawson et al., 2017), which is supported by significantly higher percentages of terpene emitters on Asia-Pacific islands. These results indicate that invasion of alien plants may lead to regional changes in biogenic terpene emissions (Harley et al., 1994; Litvak et al., 1996; Llusià et al., 2010).

Isoprenoid emissions vary greatly among plant species (Appendix S1). Emissions of isoprenoids of different species vary by several orders of magnitude (Harley et al., 1999; Kesselmeier and Staudt, 1999), especially for isoprene (Appendix S1; Figure 2A). This capacity to form and emit volatiles by a large fraction of tree species and a few herbaceous species (Appendix S1) is associated with the activity of the synthase enzyme specific for each isoprenoid (Silver and Fall, 1991; Harley et al., 2003). These rates of production catalyzed by enzymes vary widely across and within species and are influenced by a wide range of factors, such as light intensity and temperature experienced by the leaves during measurement and in the days prior to measurement, foliar age, and canopy position (Harley et al., 1999, 2003; Sharkey et al., 1999; Geron et al., 2000; Pétron et al., 2001), and even the levels of air pollutants, such as ozone (Bison et al., 2018; Yáñez-serrano et al., 2020). Therefore, these data obtained by quantitative measurements were used to identify the emitting taxa but were not robust enough to assign species-specific emission capacities, especially for those species that were characterized as emitters or non-emitters based on a single measurement or a few measurements (Harley et al., 2014). In addition, the sample plants were selected as representative of the species, but this variability within species in emission can sometimes be sufficiently large to prevent identifying potential significant differences in emissions between species. Thus, unidentified variations in emissions between species in these studies also occurred, which can be due in part to factors, such as the growth environment and/or the stage of development or even the measurement technique.

The evolutionary divergence of genes that code for enzymes of isoprenoid synthesis has been reported in narrow phylogenetic groups (Bohlmann et al., 1998; Llusià et al., 2010, 2014), but a general phylogenetic determinant for plant emission is not clear (Welter et al., 2012; Loreto and Fineschi, 2015). To the best of our knowledge, significant phylogenetic signals have been found for some individual terpene compounds, such as α-pinene (*k* = 0.651 and *p* = 0.009) in Hawaii (Llusià et al., 2010) and ocimene and γ-terpinene (*k* = 1.15 and *p* = 0.03, and *k* = 1.31 and *p* = 0.03, respectively) in Borneo (Llusià et al., 2014), indicating strong resemblance in specific terpene emissions among the most closely related species (Llusià et al., 2010) on Asia-Pacific islands and the potential of capacity to emit volatile compounds using as a basis for studying taxonomic relationships in plants (Loreto et al., 1998; Llusià et al., 2010). These relationships, however, were not ubiquitous. For example, in the above studies, we pointed out that phylogenetic signals were found only randomly and rarely among terpene compounds and were not found for total quantities of monoterpenes (*k* = 0.139 and *p* = 0.876) and total quantities of sesquiterpenes (*k* = 0.511 and *p* = 0.152) in Hawaii or total quantities of terpenes (*k* = 0.016 and *p* = 0.995) in Borneo. Moreover, we did not find this relationship for isoprene (*k* = 0.409 and *p* = 0.182) among 42 plant species in Okinawa. On the other hand, the relationship between plant species and isoprene emissions was observed in two of the most commonly studied plant families containing >40 species. Moraceae (41 species, *k* = 0.146 and *p* = 0.578) and Fabaceae (108 species, *k* = 0.110 and *p* = 0.109; Appendices S1 and S2), and the phylogenetic signals were insignificant for the two dominant families (Webb and Donoghue, 2005). A previous study reported that isoprene emissions are likely ancestral within the family Fabaceae (Monson et al., 2013), while Fabaceae harboring many strong emitters still had very low isoprene emissions in this study (Figure 2B; Appendix S1). The lower emission potentials were thus generally not due to a lack of genera with characteristically high emissions but indicated a lower emission potential within specific phylogenetic lines (Llusià et al., 2010; Monson et al., 2013), which may obscure the recognition of phylogenetic signals at the family level. Although the phylogenetic distribution of isoprene emissions broadly across the phylogeny of plants, isoprene synthase is so readily lost and gained that its signal can be difficult to distinguish from noise in the phylogeny at both large and fine phylogenetic scales (Monson et al., 2013; Loreto and Fineschi, 2015). However, the lack of phylogenetic relationships of isoprenoids in this study could be masked by the multiple factors that control their synthesis and emission (Peñuelas and Llusià, 2001).

## Seasonal and Diurnal Variations of Isoprenoid Emissions From Tropical Species

Emissions can differ greatly between trees of the same species and even between leaves from the same tree due to differences in the growth environment and/or developmental stage (Lerdau and Throop, 2000; Kuhn et al., 2004a; Wilske et al., 2007; Laffineur et al., 2011; Šimpraga et al., 2011, 2019; Alves et al., 2018). These differences can be due to diurnal and seasonal phenological cycles, including large uncertainties derived from individual plant health, status of herbivory, local soil moisture content and nutrient availability, local shading and microclimate, foliar age, and past meteorological conditions (Kesselmeier and Staudt, 1999; Geron et al., 2001; Niinemets et al., 2010; Llusià et al., 2010, 2014; Alves et al., 2018).

### Seasonal Variations

Environmental conditions, such as temperature and light intensity, generally vary seasonally less in tropical than temperate ecosystems. Primary physiology indicated by the net photosynthetic rate varied little in the Congo (Serça et al., 2001) and Amazon regions (Andreae et al., 2002; Kuhn et al., 2004a) and was not greatly affected by seasonal changes in rainfall or the amount of soil moisture (Roberts et al., 1990; Kuhn et al., 2004a; Rottenberger et al., 2004). More recent studies demonstrated significant photosynthetic seasonality, with photosynthesis increasing during the dry season in Amazon evergreen forests, and demonstrated leaf age-dependent physiology as the mechanism (Wu et al., 2016; Albert et al., 2018). In addition, lower rates of stomatal conductance and transpiration in the dry season are caused by a lower foliar water potential, leading to lower evaporative cooling and hence higher foliar temperatures (Guenther et al., 1999b).

The seasonal variations in isoprene emissions may be species-specific and have been correlated with the inherent capacity of leaves to synthesize isoprene (Monson et al., 1994; Schnitzler et al., 1997; Kuhn et al., 2004a), depending on the activity of extractable isoprene synthase (Kuzma and Fall, 1993; Lehning et al., 1999; Kuhn et al., 2004a) and/or the availability of the isoprene precursor substrate dimethylallyl diphosphate (DMAPP; Geron et al., 2000; Brüggemann and Schnitzler, 2002). These variations can be influenced by a number of physiological and environmental drivers, including the seasonal dependence of foliar maturation, changes in the growth environment of leaves, availability of water and nutrients, and even the growth conditions preceding measurement (Harley et al., 1999, 2003; Sharkey et al., 1999; Geron et al., 2000; Pétron et al., 2001; Kuhn et al., 2004b; Alves et al., 2014, 2018). The rates of isoprene emission from mature leaves of tropical plants were commonly higher and sometimes more than two-fold higher in the dry season than in the wet season (Guenther et al., 1999b; Klinger et al., 2002; Kuhn et al., 2004a; Geron et al., 2006; Wilske et al., 2007). Isoprene emissions increased during the dry season due to higher cumulative exposure to solar radiation (Sharkey et al., 1999; Geron et al., 2000, 2006; Pétron et al., 2001) and lower water availability under similar temperature conditions. This increase in the dry season is quite frequent in much of the Amazon, especially when radiation and soil water retention are high and deep roots allow sustained transpiration (Maeda et al., 2017), which is part of the mechanism for dry season greening (Huete et al., 2006) and is also linked to the emission seasonality demonstrated by Alves et al. (2018). Isoprene emissions from *Celtis philippensis* leaves in Xishuangbanna in China, however, were higher from new leaves in the wet season (September) than from old leaves of the previous dry season (April; Wilske et al., 2007). These results were in accordance with the finding that the production of isoprene decreases dramatically with foliar senescence (Harley et al., 1994; Tambunan et al., 2006), so higher rates of isoprene emission for tropical plants can be expected with the flush of new leaves compared to old leaves (Kuhn et al., 2004a,b). Isoprene emissions in Puerto Rico did not differ significantly between seasons among *Pisonia albida*, *Bursera simaruba*, *Capparis indica*, *Capparis cyanophollora,* and *Clusia rosea* (Lerdau and Keller, 1997).

In contrast to the increase in isoprene emissions, monoterpene emissions were lower in the dry season than in the wet season for *Apeiba tibourbou* at an Amazonian site (10°08′43″S, 62°54′27″W; Kuhn et al., 2004a) and for *Hevea brasiliensis* at a Xishuangbanna site (21°55′25″N, 101°16′05″E; Wang et al., 2007). The differences in emissions between the dry and wet seasons, however, were not as large at the Amazonian site than at the Xishuangbanna site, where the difference in water availability was more extreme. Although light-dependent foliar terpenes exited the plant predominantly through the stomata (Loreto et al., 1996b; Wang et al., 2007), their decrease in emission could not be explained by the lower stomatal conductance in the dry season. This tended to be offset by high biosynthesis/concentrations of intercellular terpenes, increasing the gradient of leaf-to-air terpene vapor pressure (Fall and Monson, 1992; Kuhn et al., 2004a). Low emission rates were accompanied by low rates of transpiration and photosynthesis, indicating that the canopy in the dry season may have been senescent (Wang et al., 2007). The low emissions in the dry season may be from small residual stored pools and may be masked by instantaneous emissions in the wet season, whereas the large emissions in the wet season may be mostly due to sources of rapid synthesis (not stored; Wang et al., 2007). However, Jardine et al. (2017) showed highly seasonally dynamic monoterpene emissions from an Amazon site, with total quantities of monoterpenes significantly increasing during the dry season under an El Niño drought, during which period leaves reduced transpiration and rose in temperature.

The composition of emissions from *A. tibourbou* in the Amazonian forest differed little between the dry and wet seasons (Kuhn et al., 2004a), but emissions from *H. brasiliensis* in Xishuangbanna (Wang et al., 2007) consisted of few compounds due to much lower emissions in the dry season. Monoterpene emissions for tropical species are generally dominated by only a few compounds, with sabinene, α-pinene, β-pinene, and limonene together accounting for approximately 90% of the total amounts in both seasons (Kuhn et al., 2002, 2004a; Wang et al., 2007; Wilske et al., 2007). β-ocimene was found at concentrations similar to α-pinene in an Amazon canopy study (Jardine et al., 2015) and therefore may be another dominant compound emitted by tropical species. The species had different patterns of emitted monoterpene groups, but some patterns were consistent across different tropical species: emissions of α-pinene and/or limonene were present or even higher with the flush of new leaves and decreased as the leaves aged, but myrcene and/or camphene tended to be present in the emissions of old leaves (Wilske et al., 2007). Higher α-pinene and limonene emissions in a species were also concurrent with higher foliar temperatures in one season compared to the other season (Wilske et al., 2007).

### Diurnal Variations

The diurnal pattern of isoprenoid emissions was very similar among tropical species (Kuhn et al., 2002, 2004a; Padhy and Varshney, 2005b), despite differences in emission composition and magnitude. The emissions of all isoprenoids were negligible at night, increased in the morning as the air temperature and light intensity increased, and rates were more or less maintained when emissions were highest and decreased in the afternoon (Kuhn et al., 2002, 2004a; Padhy and Varshney, 2005b; Jardine et al., 2017). Isoprene emissions were confined to the daytime only (Rinne et al., 2002; Kuhn et al., 2004a; Padhy and Varshney, 2005b) and were not affected by the decrease in stomatal conductance at midday, as reported for *Sorocea guilleminiana* in a diel course of measurements by Kuhn et al. (2004a). No emissions of monoterpenes were detected at night in species that did not store terpenes, even at high temperatures (Kuhn et al., 2004a). The emission of α-pinene, however, was variable in some species and was not correlated with either day or night (Padhy and Varshney, 2005a).

Total monoterpene emissions have a distinct diel cycle, with most emitted monoterpenes following this pattern of emissions and accounting for a similar proportion of the composition throughout the day (Kuhn et al., 2002, 2004a) within individual species; however, variability in emissions existed in compounds, such as sabinene and limonene, among species (Wang et al., 2007). Interestingly, the emission of cis-ocimene from *H. brasiliensis* had a distinct diurnal trend, with a significantly higher contribution to the total emissions at midday (Wang et al., 2007) at the beginning of the wet season. The increase and decrease in cis-ocimene emissions during the day suggests that the diurnal pattern of these emissions may be under circadian control. This circadian control might act *via* the substrate supply or the production or activity of synthases (Wilkinson et al., 2006; Loivamaki et al., 2007), indicating that tropical monoterpene emissions are expected to have environmental and physiological controls similar to those of isoprene (Kuhn et al., 2002; Jardine et al., 2017).

## Modification of the G93 Model to Predict Isoprenoid Emissions From Tropical Species

Emission inventories and model calculations have been developed to define the strength of regional and global BVOC emissions, but they rely mostly on studies of VOC emissions from temperate areas of North America and Europe, and more efforts in parameterizations are warranted for tropical regions (Guenther et al., 1996b, 1999b, 2012; Holm et al., 2014; Mutanda et al., 2016; Higa et al., 2018).

### Description of the G93 Model for Isoprene Emission

The G93 model, with its further developments, is the most widely used model for emissions of isoprene and other BVOCs from plants (Guenther et al., 1993, 2006, 2020). It simulates instantaneous emission rates as a product of the response functions for light and temperature after adjustment by a species-specific emission factor. Foliar temperature and light intensity are the primary drivers of vegetation emissions. Its further developments, such as G99 (Guenther et al., 1999b) and MEGAN (Guenther et al., 2006), were expressed as functions of more drivers, for example, the influence of leaf age and the past conditions of light intensity and temperature experienced by the leaves were considered. They were used to model the landscape/ecosystem foliar emissions into the above-canopy atmosphere.

Different compounds do not necessarily follow the G93 algorithm, especially where the compounds are controlled by environmental parameters other than light and temperature and other circadian controls, but G93 is still the most important model for describing biogenic isoprenoid emissions due to its extensive and indiscriminate use in predicting isoprene emissions from plants in both temperate and tropical regions (Benjamin et al., 1996; He et al., 2000; Owen et al., 2001; Harley et al., 2004; Kuhn et al., 2004a,b; Padhy and Varshney, 2005a). The performance of G93 is satisfactory for predicting the rate of isoprene emission from temperate species but possibly not for estimating the rate from tropical species, with studies already negating the possibility that the observations may have been due to differences in method or environmental conditions, suggesting that emission responses may differ between tropical and temperate plants (Kuhn et al., 2004a,b; Guenther et al., 2006; Bracho-Nunez et al., 2013).

The G93 model estimates isoprene emissions (*I*) as a function of two environmental drivers, temperature and light intensity:


(1) $$I=Is.C_{T}.C_{L}$$

where *I* is the predicted emission rate at temperature *T* (K) and photosynthetically active radiation (PAR) of *L* (μmol m^−2^ s^−1^) and *Is* is the basal emission rate (emission factor). *C*_*T*_ and *C*_*L*_ are coefficients for temperature and light intensity, respectively, and are defined by:


(2) $$C_{T}=\left\{exp\left[C_{T1}\left(T-Ts\right){\left(RT_{S}T\right)}^{-1}\right]\right\}{\left\{1+exp\left[C_{T2}\left(T-Tm\right){\left(RTsT\right)}^{-1}\right]\right\}}^{-1}$$

(3) $$C_{L}=(\alpha C_{L1}L){\left(1+\alpha^{2}L^{2}\right)}^{-0.5}$$

where *C*_*T*1_ = 95,000 J mol^−1^, *C*_*T*2_ = 230,000 J mol^−1^, *Tm* = 314 K, *α* = 0.0027, *C*_*L*1_ = 1.066, *R* = 8.314 J K^−1^ mol^−1^ and *Ts* is the foliar temperature under standard conditions (303 K, Guenther et al., 1993).

### Modification of the G93 Model to Predict Isoprene Emissions From Tropical Species

Isoprene emissions for tropical species increased exponentially with temperatures up to 40°C (Keller and Lerdau, 1999; Lerdau and Throop, 1999; Harley et al., 2004) and stabilized at higher temperatures than for temperate species, sometimes even >45°C (Alves et al., 2014). This difference could be due to differences in the growth conditions (Monson et al., 1992), so the optimal temperature for isoprene emission by tropical species may vary considerably. *C*_*T*_ for G93 postulates that the maximal temperature-dependent isoprene emission is almost 2-fold higher than the basal emission rate (*Is*). This magnitude is between 2 and 4 for most temperate plants (Monson et al., 1992; Sharkey and Monson, 2014) but is >5 for most tropical tree species and > 10 for the regional highest emitters (*Ficus virgata* and *Calophyllum inophyllum*; Oku et al., 2008, 2014; Higa et al., 2018). The pattern of the response of isoprene emissions to temperature for tropical species thus notably differed from the pattern predicted by the G93 model. In addition, the actual relative emission rate under standard conditions was notably lower than that postulated by the G93 model, which tended to overestimate *Is* when the average *Is* was estimated by G93 under variable conditions of temperature and light intensities for tropical species (Oku et al., 2008). Therefore, G93 may provide higher normalized emission rates under standard conditions but lower rates for predicted emissions under high temperatures and light intensities, which also tended to overestimate the emission rate in the morning and evening and to underestimate the rate at midday (Oku et al., 2008; Higa et al., 2018).

Some tropical species were not light saturated for isoprene emission up to a photosynthetic photon flux density (PPFD) of 2,000 μmol m^−2^ s^−1^ in the Republic of Panama (Keller and Lerdau, 1999; Lerdau and Throop, 1999), Puerto Rico (Lerdau and Keller, 1997) and Brazil (Alves et al., 2014). Harley et al. (2004), however, reported isoprene light saturation at a PPFD of 1,500 μmol m^−2^ s^−1^ for *Mangifera indica*, and Kuhn et al. (2004a) reported isoprene light saturation at PPFDs <1,000 μmol m^−2^ s^−1^ for *Hymenaea courbaril*. The emission rate for *F. virgata* in Okinawa normalized to temperature increased linearly with light intensity up to 1,700 μmol m^−2^ s^−1^ in a phytotron (Tambunan et al., 2006), did not saturate up to 1,800 μmol m^−2^ s^−1^ in the field (Tambunan et al., 2006) and stabilized at light intensities >1,200 μmol m^−2^ s^−1^ in plastic pots (Oku et al., 2008) under natural conditions. Light saturation for isoprene emissions may therefore vary among tropical species and even for the same species under different growth conditions. Isoprene emissions for at least some tropical taxa did not saturate at light intensities under natural conditions. Foliar temperature was well correlated with light intensity, but the differences in *C*_*L*_ between plant species were smaller at higher temperatures; the poorer performance of G93 for high emission rates at high temperatures for tropical trees was therefore mostly due to the low efficiencies of *C*_*T*_ in G93 (Oku et al., 2008, 2014; Mutanda et al., 2016). These observations strongly indicate that the general parameterization of G93 is not suitable for tropical trees, corroborating findings from Africa where specific modifications of parameters and seasonality were suggested (Guenther et al., 1999b).

Harley et al. (2004) used the temperature- and light-dependent algorithm of G99 to estimate foliar emissions of *M. indica*. The light response of isoprene emission (*E_L_*) is thus calculated in dependence on PPFD (μmol m^−2^ s^−1^) according to:


(4) $$E_{L}=Es\left(\alpha C_{L}PPFD\right)/[{\left(1+\alpha^{2}PPFD^{2}\right)}^{0.5}]$$

where *Es* is the emission rate under standard conditions (emission factor), and *α* and *C*_*L*_ are empirical coefficients.

The temperature response of isoprene emissions (*E*_*T*_) is accordingly represented in dependence on leaf temperature (*T*_*L*_, K) as:


(5) $$E_{T}=E_{opt}C_{T2\;}exp\left(C_{T1}x\right)/\{C_{T2}-C_{T1}[1-exp(C_{T2}x)]\}$$

where *x = [(1/T_*opt*_) − (1/T_*L*_)]/R*, *R* is the gas constant (0.008314 kJ K^−1^ mol^−1^), *T*_*opt*_ is the temperature optimum (K), *E*_*opt*_ is the emission rate (μg C g^−1^ h^−1^) at *T*_*opt*_, and *C*_*T*1_ and *C*_*T*2_ are empirical coefficients representing the energies of activation and deactivation, respectively (kJ mol^−1^). *α* and *C*_*L*_ in Eq. (4), and *C*_*T*1_ and *C*_*T*2_ in Eq. (5) are determined by the non-linear best fit using a non-linear least squares regression routine (KaleidaGraph, Synergy Software; Harley et al., 2004).

Another approach to define optimized parameterization of the G93 algorithm without changing anything of the model as such has been introduced as the iterative “Ping-Pong” method (Mutanda et al., 2016). This process was successfully used to simultaneously define parameters *C*_*T*1_ and *C*_*T*2_ in Eq. (2), and *α* in Eq. (3) (Mutanda et al., 2016; Higa et al., 2018). For illustration, original and changed parameters are presented in Table 1 for the species applied in these two studies. This table shows that temperature-related parameters for all tropical species were found to be approximately 1.5 (*C*_*T*2_)- and 2 (*C*_*T*1_)-fold those of the original approach. Also, the procedure resulted in a generally higher sensitivity to light (up to 4.5-fold higher α).

**Table 1:** G93 parameters for various tropical species as obtained from optimization in Mutanda et al. (2016) and Higa et al. (2018).

Species	Optimized G93				G93	Reference
	*C_T1_*	*C_T2_*	*α*	M score	M score	
*Casuarin equisetifolia*	187,125 ± 14,570	151,300 ± 46,881	0.0052 ± 0.0008	0.011 ± 0.003	0.089 ± 0.018	Mutanda et al. (2016)**
*Ficus septica*	158,500 ± 15,471	287,250 ± 94,715	0.0034 ± 0.0006	0.105 ± 0.029	0.205 ± 0.033	Mutanda et al. (2016)
*Bauhiniav ariegata*	173,500 ± 13,714	310,500 ± 25,423	0.0019 ± 0.0003	0.023 ± 0.007	0.070 ± 0.013	Higa et al., 2018
*Calophyllum inophyllum*	222,000 ± 20,251	386,833 ± 54,449	0.0117 ± 0.0085	0.069 ± 0.020	0.284 ± 0.042	Higa et al., 2018
*Garcinia subelliptica*	208,750 ± 13,278	342,500 ± 66,666	0.0097 ± 0.0048	0.026 ± 0.007	0.136 ± 0.027	Higa et al., 2018
*Mangifera indica (red)*	168,333 ± 7,339	303,833 ± 62,414	0.0116 ± 0.0033	0.007 ± 0.002	0.045 ± 0.011	Higa et al., 2018
*Mangifera indica (yellow)*	181,167 ± 3,919	303,667 ± 34,108	0.0063 ± 0.0025	0.015 ± 0.004	0.066 ± 0.012	Higa et al., 2018
*Syzygium cumini*	193,833 ± 9,821	365,500 ± 21,465	0.0030 ± 0.0002	0.025 ± 0.006	0.091 ± 0.018	Higa et al., 2018
*Syzygium samarangense*	193,333 ± 2,186	385,333 ± 15,423	0.0049 ± 0.0014	0.009 ± 0.002	0.077 ± 0.015	Higa et al., 2018

Normalized mean square error (M score) is given for the optimized and standard parameterization in G93 (C_T1_ = 95,000, C_T2_ = 230,000, *α* = 0.0027). Performance values are means from *n* = 3–4.

Besides the modifications in parameterization of G93, supplementary formulas were also proposed to predict isoprene emissions from some tropical species. Keller and Lerdau (1999) proposed a simple formula (Eq. (6)) as an alternative to the light response formula (Eq. (3)) of G93 to account for the lack of light saturation in tropical species. Oku et al. (2008) modified the formula for calculating the factor *C*_*L*_ (Eq. (7)) to incorporate a decrease in the observed rate of isoprene emission from *F. virgata* at high light intensity.


(6) $$C_{L}={\left(aL\right)}^{b}$$

where *a* = 0.001 μmol m^−2^ s^−1^, the non-linear best fit subject to the constraint that *C*_*L*_ equal unity at PAR of 1,000 μmol m^−2^ s^−1^, was achieved with *b* = 0.3460 for 12 tropical species investigated (Keller and Lerdau, 1999).


(7) $$C_{L}=(\alpha C_{L1}L)\;{\left(1+\alpha^{2}L^{2}\right)}^{-0.5}{\left(1+e^{\left(\beta +\gamma L\right)}\right)}^{-1}$$

where *β* and *γ* are new empirical coefficients determined by the non-linear best fit (Oku et al., 2008).

### Description and Modification of the G93 Model for Monoterpenes

In contrast to isoprene, however, monoterpene-producing plants mostly accumulate pools of monoterpenes and store them in specialized structures, such as resin ducts, glandular trichomes, or similar structures (Monson et al., 1995; Kesselmeier and Staudt, 1999; Kuhn et al., 2002, 2004a). Monoterpene emissions are often regarded as originating from storage only. In this case, the process can be described as temperature-dependent with a simple exponential equation, as also suggests the G93 model:


(8) $$M=Ms.exp\left(\beta \left(T-Ts\right)\right)$$

where *M* is the rate of monoterpene emission at temperature *T* (K), *Ms* is the rate of monoterpene emission at a standard temperature *Ts* (K). *β* (K^*−*1^) is an empirical coefficient that establishes the dependence on temperature of the emission rate in the equation. This *β* (K^*−*1^) varies between monoterpenes with different vapor pressures and solubilities, and plants with different storage and emission pathways (Guenther et al., 1993). Guenther et al. (1993) indicated that the estimates of *β* varied from 0.057 to 0.144 K^*−*1^, with about half the estimates falling within the range of 0.09 ± 0.015 K^*−*1^ among all 28 estimates collected. Eq. (8) was typically used to simulate the dependence on temperature of the rate of monoterpene emission (Lamb et al., 1987; Pierce and Waldruff, 1991; Roselle et al., 1991). It usually works for terpene-storing species for which changes in relative humidity and light intensity have a negligible impact on short-term variations in monoterpene emissions (Juuti et al., 1990; Guenther et al., 1991).

It is, however, known that some species emit monoterpenes directly from production without hosting any specific terpene storages (Staudt and Seufert, 1995; Staudt and Bertin, 1998; Owen et al., 2002; Dindorf et al., 2006). At an Amazonian site, monoterpene emission from *A. tibourbou* showed the same dependence on light as isoprene emission, with no monoterpenes released under dark conditions and with an increase and saturation at low and higher light intensities, respectively (Kuhn et al., 2002, 2004a). Similarly, the emissions of sabinene, α-pinene, and β-pinene from *H. brasiliensis* at a Xishuangbanna site, in response to temperature were very similar to those of isoprene emissions (Guenther et al., 1993), although the maximum temperature was lower than normal for isoprene emissions from leaves (Geron et al., 2006). This indicates that monoterpene emissions may be under similar physiological controls as light-dependent isoprene emissions for some tropical species. In addition, Jardine et al. (2015) found the light dependence of monoterpene emissions from seven tree species across the Amazon Basin by conducting light curves under constant temperature and CO_2_. For example, measurements on the leaves of a common pioneer species *Cecropia sciadophylla* showed light-dependent emissions of six monoterpenes, with the dominance of trans-β-ocimene and cis-β-ocimene in the light-dependent emissions.

Monoterpenes are formed from photosynthetic intermediates and may share the same synthetic pathway with isoprene within plant plastids (Loreto et al., 1996a; Kuhn et al., 2002), so monoterpene synthesis may also be assumed to depend on light intensity, supported also by widely reported canopy scale light-dependent emissions of monoterpenes from Amazonian rainforest (Rinne et al., 2002; Karl et al., 2007; Yàñez-Serrano et al., 2015, 2018; Jardine et al., 2017). For species that lack specific storage pools, the isoprene algorithm of G93 consequently fits the observed data of their monoterpene emissions well because monoterpene emissions were modeled analogously to isoprene emissions (Kuhn et al., 2002; Wang et al., 2007). We suggest that a strong dependence of monoterpene emissions on light intensity may be widespread among tropical tree species, which has a strong impact on the magnitude and distribution of both past analysis and future prediction for these compounds (Rinne et al., 2002; Kuhn et al., 2004a; Jardine et al., 2015).

### Potential Modification of the G93 Model to Predict Seasonal Variations in Isoprenoid Emissions

The G93 model may not fully account for seasonal variations because these long-term differences in rates of isoprenoid emission cannot be reconciled solely with instantaneous meteorological data of light intensity and temperature (Yokouchi and Ambe, 1984; Alves et al., 2018). The influence of seasonality is important in Amazonia (Kuhn et al., 2002) and Xishuangbanna (Wang et al., 2007). Other important tropical regions (e.g., Borneo), however, have little or no climatic seasonality, so emission algorithms need to be more region-specific (Higa et al., 2018). Isoprene emission from tropical plants is also associated with physiological and developmental changes during the growing season, the capacity of isoprene emission to vary with foliar age and the highest capacity of isoprene emission from young mature leaves (recently fully expanded; Kuhn et al., 2004b; Alves et al., 2014), indicating a variable inherent capacity of plants to synthesize isoprene during foliar development. Foliar physiological activity associated with isoprene emissions depends on the ontogenetic stage of the leaves, increasing rapidly in developing leaves and decreasing in senescing leaves due to programmed cell death (Niinemets et al., 2004; Alves et al., 2014).

In addition to the changes in isoprene emission capacity, Alves et al. (2014) also found that light saturation for isoprene emission could also vary with foliar age, demonstrating that biological changes due to aging could also influence the pattern of the response of isoprene emission to light. These results, however, disagreed with those by Kuhn et al. (2004a), who stated that the inherent capacity of a plant to synthesize isoprene varied without modifying the function of the light response during foliar development. Alves et al. (2018) demonstrated that characterizing the isoprene emission capacity throughout the seasons based on the leaf area index and phenology improved the model observation agreement. More studies are needed to elucidate how the models can be modified to identify the effects of foliar phenology on isoprenoid emissions.

## Techniques for Sampling and Measuring Isoprenoid Emissions From Tropical Species

Enclosed measurement systems, initially developed by Zimmerman (1979), are the most commonly used sampling method for studying BVOCs from tropical plants (Guenther et al., 1996a Greenberg et al., 2003; Harley et al., 2003, 2004; Padhy and Varshney, 2005a; Tambunan et al., 2006; Singh et al., 2008, 2011; Niinemets et al., 2011; Malik et al., 2018, 2019; Zeng et al., 2022). Enclosure, however, may underestimate emissions due to foliar shading (Guenther et al., 1996a) and contribute erratic emissions to α-pinene (Padhy and Varshney, 2005b). LI-6400 (LI-COR, Inc., Lincoln, NE, United States; Keller and Lerdau, 1999; Lerdau and Throop, 1999; Geron et al., 2002, 2006; Alves et al., 2014; Jardine et al., 2016) and ADC (LCpro, ADC BioScientific Ltd., Hoddesdon, United Kingdom; Geron et al., 2006; Llusià et al., 2010, 2014) gas-exchange systems have also been widely used for sampling VOC emissions. In addition, Taylor et al. (2021) introduced a new field instrument called “PORCO” based on photoionization of organic compounds for measuring emissions in the tropics.

BVOCs can also be characterized and quantified using different methods. Sensible choice and collocation in analytical adaptations can facilitate their quantification (Merfort, 2002; Helmig et al., 2004; Tholl et al., 2006; Bracho-Nunez et al., 2013) and enable a broad range of BVOCs to be determined (Guenther et al., 1999a; Bracho-Nunez et al., 2013). A portable photoionization detector (PID) was used in flow-through mode to qualitatively identify VOC emitters (Klinger, 1998) and semi-quantitatively assign *in situ* emission potentials as high, moderate, or low (Geron et al., 1994; Klinger et al., 2002). VOC emissions have also been quantified using a Photovac Voyager portable gas chromatograph (Lerdau and Throop, 1999; Geron et al., 2002; Harley et al., 2003). Proton transfer reaction-mass spectrometry (PTR-MS) for detecting all VOCs with proton affinities higher than water has facilitated the study of short-chain oxygenated VOCs (Bracho-Nunez et al., 2013). Other studies, however, have confirmed the difficulty of using PTR-MS for measuring monoterpene and sesquiterpene emissions, reporting high fragmentation patterns (Tani et al., 2003; Demarcke et al., 2009; Bracho-Nunez et al., 2011, 2013). Gas chromatography with a flame ionization detector (GC-FID) has been widely used for measuring isoprene emissions (Varshney and Singh, 2003; Padhy and Varshney, 2005b; Singh and Varshney, 2006; Singh et al., 2007; Bracho-Nunez et al., 2013; Malik et al., 2018) and is more sensitive than PTR-MS for measuring sesquiterpene emissions (Bracho-Nunez et al., 2013). GC-FID, however, misses some VOCs, such as α-terpinene, due to its low performance in separation/identification (Wilske et al., 2007). Methods using a gas chromatograph coupled to a mass spectrometer (GC–MS) are more specific than PTR-MS methods (Guenther et al., 1996a Bracho-Nunez et al., 2011) and are suitable for the determination of heavier VOCs, including sesquiterpenes (Otter et al., 2002; Bracho-Nunez et al., 2013).

## Conclusion

This review contributes to the global database of BVOC emissions from tropical plants (Appendix S1). Such emissions may represent local features due to the high biodiversity of most tropical ecosystems. Future field observations of tropical species may ultimately allow the estimation of average ecosystem emissions that could then be scaled to estimate regional emissions. More long-term measurements are needed to better characterize seasonal and interannual variability for estimating the present and future impacts of BVOC fluxes, and more attention should be given to sesquiterpenes, whose emissions are rarely studied but are highly variable and uncertain, perhaps due to methodological limitations and the inherent association of sesquiterpenes with stress and phenology (Bracho-Nunez et al., 2011, 2012). A better understanding of the behavior of BVOC emissions from the screening of additional plant species will increase our confidence in predictive models, so more experimental information is needed on how the capacities of species-specific isoprene emissions vary during foliar developmental stages to improve models that predict emissions from tropical species (Alves et al., 2014). Many uncertainties in emission models based on poor simulations of foliar-scale emissions, among other factors, however, remain (Mutanda et al., 2016). Future investigations of more possible sources are warranted, including examining the role of biodiversity and plant competition, comparing different sampling and analytical techniques (Bracho-Nunez et al., 2013), or creating techniques that encompass most of the BVOC emissions.

## Author Contributions

JP, JL, and XW conceived the idea and revised the manuscript. ZM drafted the manuscript. JZ, YZ, DA, KY, and ZY reviewed and edited the manuscript. All authors contributed to the article and approved the submitted version.

## Funding

This study was supported by the Natural Science Foundation of China (project nos. 42022023 and 41961144029), the Chinese Academy of Sciences (QYZDJ-SSW-DQC032), the Hong Kong Research Grants Council (T24-504/17-N), the Department of Science and Technology of Guangdong Province (2020B1212060053, 2017BT01Z134, and 2019B121205006), the Spanish Government project PID2019-110521GB-I00, the Catalan Government project SGR2017-1005, and the Fundación Ramón Areces grant ELEMENTAL-CLIMATE. ZM also acknowledges the financial support from the China Scholarship Council and the Guangdong Provincial Postdoctoral Talent-Introduction Program.

## Conflict of Interest

The authors declare that the research was conducted in the absence of any commercial or financial relationships that could be construed as a potential conflict of interest.

## Publisher’s Note

All claims expressed in this article are solely those of the authors and do not necessarily represent those of their affiliated organizations, or those of the publisher, the editors and the reviewers. Any product that may be evaluated in this article, or claim that may be made by its manufacturer, is not guaranteed or endorsed by the publisher.

## Supplementary Material

Appendix S1 Isoprenoid emissions for the tropical plants in this study. Rates of isoprene and terpene emissions with more than three species for tropical plant families. Isoprene emissions from plant species of the family Moraceae and Fabaceae in phylogenetic trees.

Appendix S2 Phylogenetic tree of species of the family Moraceae and Fabaceae.
