# Supplementary material for: An Overview of the Isoprenoid Emissions From Tropical Plant Species
Source: Front Plant Sci. 2022 May 20;13:833030. doi: 10.3389/fpls.2022.833030 (PMC9163954; doi:10.3389/fpls.2022.833030)
Supplement: Appendix S2 — Phylogenetic tree of species of the family Moraceae and Fabaceae. [file Data_Sheet_1.docx]

(a)
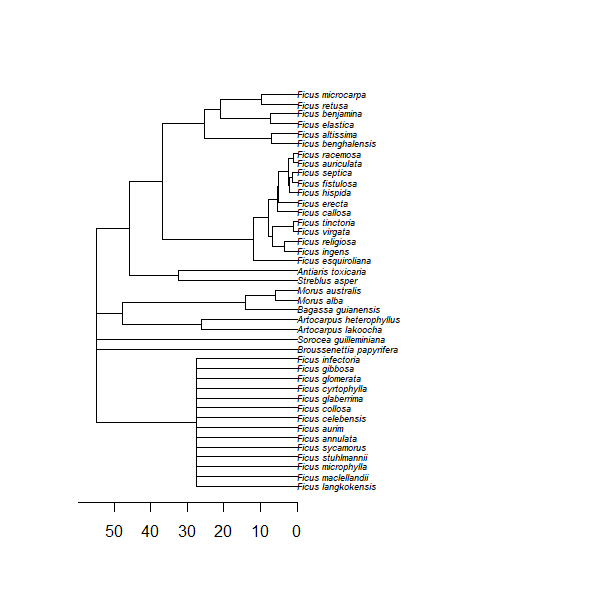


(b)


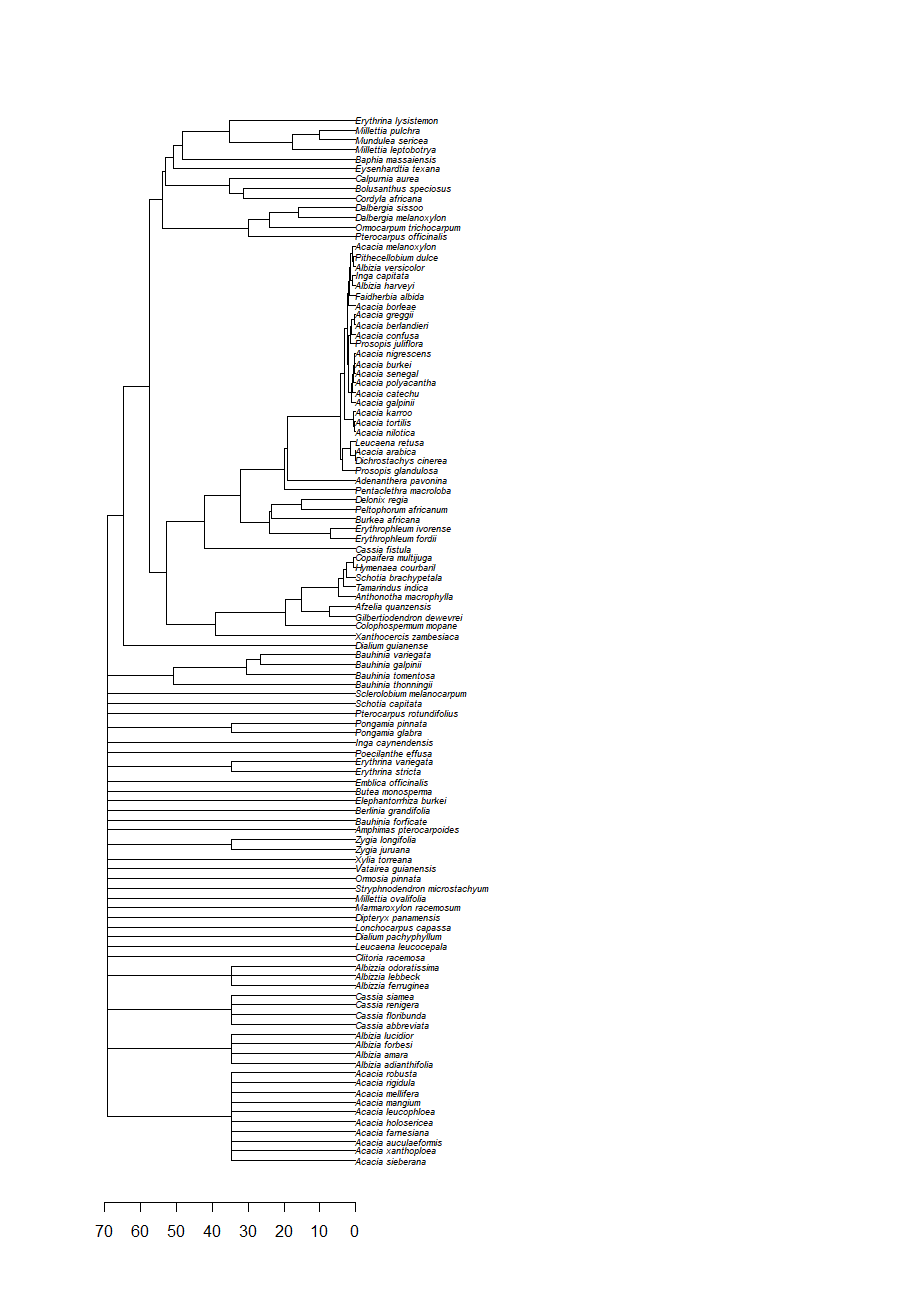


Phylogenetic tree of species of the family Moraceae (a) and Fabaceae (b), obtained using Phylomatic (Webb and Donoghue, 2005). The scale depicts millions of years.
