# Supplementary material for: An Overview of the Isoprenoid Emissions From Tropical Plant Species
Source: Front Plant Sci. 2022 May 20;13:833030. doi: 10.3389/fpls.2022.833030 (PMC9163954; doi:10.3389/fpls.2022.833030)
Supplement: Supplementary file 3 [file Table_1.docx]

**TABLE 1** G93 parameters for various tropical species as obtained from optimization in Mutanda et al. (2016) and Higa et al. (2018). Normalized mean square error (M score) is given for the optimized and standard parameterization in G93 (*C_T1_* = 95000, *C_T2_* = 230000, *α* = 0.0027). Performance values are means from n = 3–4.

| Species | Optimized G93 | | | |  | G93 | Reference |
| --- | --- | --- | --- | --- | --- | --- | --- |
|  | *C_T1_* | *C_T2_* | *α* | M score |  | M score |  |
| *Casuarin equisetifolia* | 187125 ± 14570 | 151300 ± 46881 | 0.0052 ± 0.0008 | 0.011 ± 0.003 |  | 0.089 ± 0.018 | Muntanda et al. (2016) |
| *Ficus septica* | 158500 ± 15471 | 287250 ± 94715 | 0.0034 ± 0.0006 | 0.105 ± 0.029 |  | 0.205 ± 0.033 | Muntanda et al. (2016) |
| *Bauhiniav ariegata* | 173500 ± 13714 | 310500 ± 25423 | 0.0019 ± 0.0003 | 0.023 ± 0.007 |  | 0.070 ± 0.013 | Higa et al. (2018) |
| *Calophyllum inophyllum* | 222000 ± 20251 | 386833 ± 54449 | 0.0117 ± 0.0085 | 0.069 ± 0.020 |  | 0.284 ± 0.042 | Higa et al. (2018) |
| *Garcinia subelliptica* | 208750 ± 13278 | 342500 ± 66666 | 0.0097 ± 0.0048 | 0.026 ± 0.007 |  | 0.136 ± 0.027 | Higa et al. (2018) |
| *Mangifera indica (red)* | 168333 ± 7339 | 303833 ± 62414 | 0.0116 ± 0.0033 | 0.007 ± 0.002 |  | 0.045 ± 0.011 | Higa et al. (2018) |
| *Mangifera indica (yellow)* | 181167 ± 3919 | 303667 ± 34108 | 0.0063 ± 0.0025 | 0.015 ± 0.004 |  | 0.066 ± 0.012 | Higa et al. (2018) |
| *Syzygium cumini* | 193833 ± 9821 | 365500 ± 21465 | 0.0030 ± 0.0002 | 0.025 ± 0.006 |  | 0.091 ± 0.018 | Higa et al. (2018) |
| *Syzygium samarangense* | 193333 ± 2186 | 385333 ± 15423 | 0.0049 ± 0.0014 | 0.009 ± 0.002 |  | 0.077 ± 0.015 | Higa et al. (2018) |
